# Multi-objective steel plate cutting optimization problem based on real number coding genetic algorithm

**DOI:** 10.1038/s41598-022-27100-2

**Published:** 2022-12-28

**Authors:** Jianqiao Xu, Wenguo Yang

**Affiliations:** 1 School of Pharmacy, Nanjing University of Chinese Medicine, Nanjing, Jiangsu Province China; 2 School of Artificial Intelligence and Information Technology, Nanjing University of Chinese Medicine, Nanjing, Jiangsu Province China

**Keywords:** Engineering, Mathematics and computing

## Abstract

The rectangular packing problem is an NP-complete combinatorial optimization problem. This problem occurs widely in social production scenarios, with steel plate cutting being one example. The cutting scheme for the rectangular packing problem needs to be improved because, without the globally optimal solution, there are many unnecessary edges in the steel cutting process. Based on a practical roll-fed disc shearing steel plate optimization problem, this paper explores a generalized packing method for rectangles of special dimensions and abstractly condenses complex quantitative relationships to establish a multi-objective mixed-integer nonlinear programming model. An innovative algorithm design based on a genetic algorithm is established to plan the cutting scheme in a high-speed and efficient way. The outcome is a utilization rate of up to 92.73% for raw materials and a significant reduction in labor, providing a guide for practical production and processing tasks. The advantages and disadvantages of the model and algorithm are discussed, and it is concluded that this rectangular packing method has strong universality and generalization ability, allowing rectangular packing tasks with large data volumes to be completed within a short time.

## Introduction

The rectangular packing problem^1^ is known to be NP-complete^2,3^. The core task of the rectangular packing problem is to place a number of small rectangles in a large rectangular frame as efficiently as possible. In social production, the cutting of steel plates and wooden boards are typical rectangular packing problems. However, in the actual production process, the producer often simply arranges the sample by observation and measurement, resulting in a large amount of wasted material and unnecessary cuts. Steel mills effectively waste more than $300 for every ton of scrap steel they produce. If a specific mathematical model could be established on the basis of known material parameters and quantities, the global planning of a steel cutting production task would greatly reduce the production of scrap steel and manpower.

The problem of rectangular pattern arrangement has been extensively studied with the goal of maximizing the utilization of the rectangular outer frame. However, in industrial rectangular cutting operations, we are not only concerned with the utilization of the raw material, but also with the complexity of the cutting process. A dense arrangement of rectangles can easily lead to an overly complex cutting process, in direct conflict with the actual needs of production. Therefore, the rectangular cutting problem is a multi-objective optimization task in which the scheduling method must be redesigned for the actual problem. Previous research on rectangular piece cutting has focused on moderate sizes and relatively little data. Cases involving long rectangles and large data volumes are significantly more difficult. This study considers the practical problem of steel coil cutting, and develops a method of plate-cutting applicable to rectangular pieces of any size along with the corresponding mathematical model.

Due to the computational complexity of NP-complete problems, it is almost impossible to find a global solution to the mathematical model. The grid search method is computationally intensive, while stochastic simulation methods produce poor optimization results and often require intelligent optimization. As our study is based on a real production problem, the mathematical model is somewhat complex as it considers the large number of constraints faced in actual production tasks. Intelligent optimization algorithms are generally poor at solving optimization tasks with a large number of constraints. Our research includes targeted modifications to genetic algorithms and application strategies to specific problems and models. This improvement strategy is applicable to any type of downstream production problem and has good generalization implications.

## Literature review

**Figure 1:**
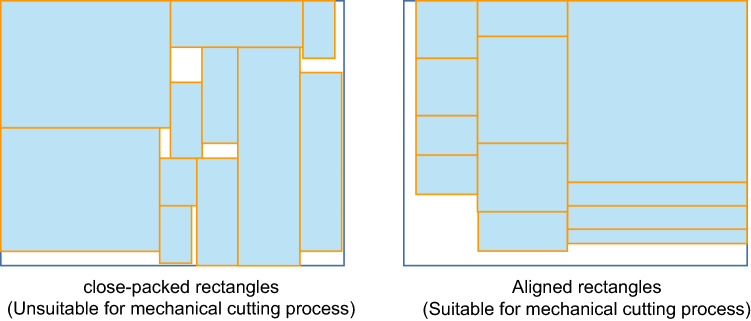
Dense rectangular arrangement (left); rectangular arrangement suitable for sawing machine (right).

Research on the rectangular packing problem is quite mature. Cid-Garcia et al.^4^ presented a two-stage positions and covering (P&C) method for two-dimensional bin packing problems. P&C arranges small rectangular pieces in a large rectangular frame through a simple two-stage algorithm, achieving a better utilization of space. Zhou et al. developed three different mathematical models^5^ for a realistic rectangular block scheduling problem based on the theory of scheduling methods, and compared the computational efficiency and performance of the different models. However, their study is mostly based on the densest packing. In the case of rectangular part-cutting problems, this may lead to an overly complex cutting process, which can only be achieved by laser cutting machines and is not applicable to mechanical cutting (see Fig. 1). In mechanical cutting, it is generally difficult to control the blade to stop at a certain shear position, and a “guillotine cut” (i.e., a shear that completely cuts off the product from one direction) method is more in line with the actual process.

**Figure 2:**
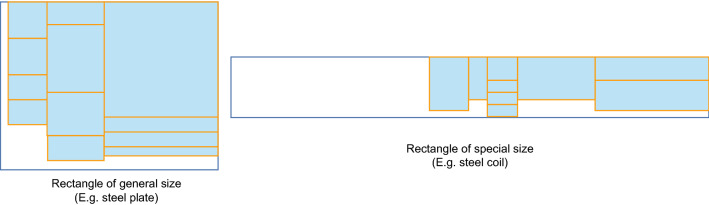
Rectangular packing problems for normal and extreme sizes.

Massberg et al.^6^studied a rectangular packing problem with additional conditions similar to guillotine constraints, but their method is not very efficient for rectangular components. Bui et al.^7^studied three practical problems of rectangular land allocation with the guillotine constraint, but their approach is not suitable for the large amounts of data typical of industrial production problems. Iori et al.^8^ split the two-dimensional packing problem into two one-dimensional arrangements by first placing one-dimensional rectangles into “strips” of equal (as much as possible) heights, and then packing the strips on the rectangular original for a second one-dimensional packing. This method is in line with the guillotine cut constraint to some extent. However, the application of this method is not very convenient for the case of steel cutting (see Fig. 2).

There have been various attempts to improve the solution efficiency, such as simulation algorithm^9^, heuristic algorithm^10,11^, improved genetic algorithm^12^, improved annealing algorithm^13^ and so on. However, intelligent optimization algorithms struggle with problems that have complex constraints. Actual production problems often have numerous complex constraints and require problem-specific analysis. In this paper, we develop a multi-objective mixed-integer nonlinear programming model for the practical example of optimizing a steel plate cutting scheme for roller disc shears. We also explore the application of innovative genetic algorithms to complex constrained production tasks.

## Problem description

**Figure 3:**
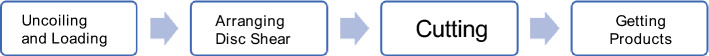
Steel cutting process.

**Figure 4:**
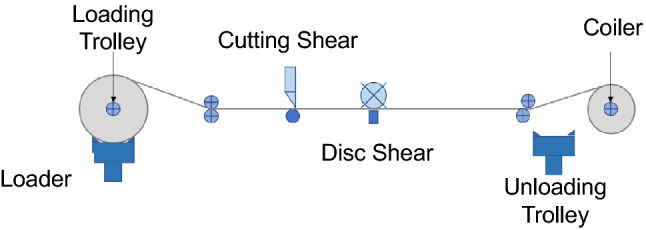
Diagram of steel operation area.

The steel cutting process used by existing roller feeding disc shears is shown in Fig. 3. The uncoiling and loading process is carried out by placing the raw steel coils on the uncoiler, unrolling and flattening them, and sending them to the operating area (see Fig. 4). The shearing process is completed on the shearing table, which in turn has a cutting head shear and a disc shear.

**Figure 5:**
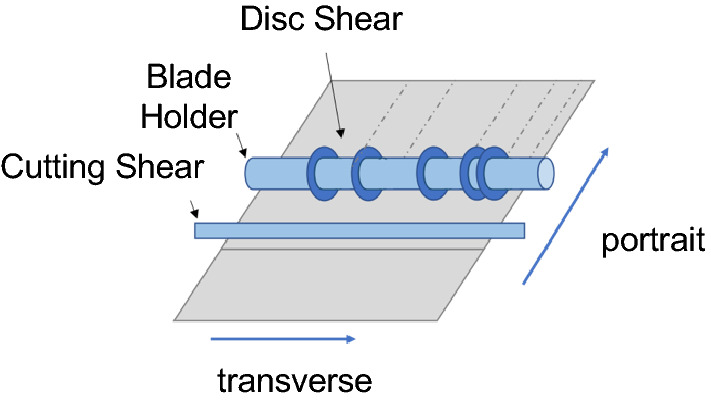
Schematic diagram of disc shear.

**Figure 6:**
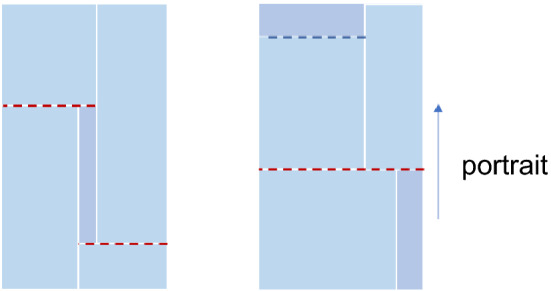
Transverse cutting schematic.

The disc shear (see Fig. 5) continuously cuts the raw material in the longitudinal direction with a rotating disc blade. Before the disc shear begins to cut, the blades must be lined up according to the ordered cutting plan. It is assumed that the blades can be arranged at any spacing on the blade holder, but the number of blades is limited to a maximum of five. Cutting with the same blade arrangement plan is called a group of orders, whereas cutting different groups requires workers to re-arrange the blades, called a blade change. Cutting each roll of raw material requires a blade change. Using the cutting shear is called a “one-cut,” which means that the whole steel plate is completely cut in the transverse direction. The one-cut requirement cannot be achieved without the cutting shear. The transverse cut on the right side of Fig. 6 (red dashed line) illustrates the one-cut scenario.

After transverse cutting, some orders may need an additional transverse cut to separate them from the scrap. These orders are moved to a smaller machine and cut again (right side of Fig. 6, blue dashed line). The same orders within the same group can be turned into finished products at the same time with one cut. There are two types of steel cutting orders: sheet orders and coil orders. The cut sheet orders are packed directly into the warehouse via the unloading trolley, while the coils need to be formed by the crimping arm of the coiler before being put into the warehouse.

If there are any leftover raw materials, we need to judge whether they meet the criteria of recyclability (**A.** length > 2000 mm and width > 1000 mm, **B.** length > 50,000 mm and width > 100 mm). If so, the residue is placed into storage for the next cutting. Leftover material that does not meet the criteria for recycling is called scrap and must be discarded. The material utilization ratio is defined as the ratio of the area cut into finished products plus the area of residual material that can be returned to storage to the total area of raw material used.

We assume that the demand for a future batch of orders is given by the factory and that the order demand quantity must be exactly met (no oversupply is allowed). Based on the existing material cutting orders in the factory, the quantity of each material used must not exceed its inventory. The disc shear requires a manual change of position on the blade holder each time the blades are scheduled, as well as a human intervention if the material needs to be moved to the small machine for re-cutting. To reduce labor costs, we wish to minimize the number of blade changes and the number of cuts on the small machine.

When an order is specified with a float percentage, the delivered order length can be varied. For example, if a float percentage of 5% is specified, the cut length can be between 95 and 105% of the original length.

In contrast to the general rectangular packing problem, the raw material used in this practical problem is a steel coil, which is characterized by a large area and long length. As a result, the computational load increases significantly, and so most rectangular packing methods are not applicable to this material. The size and quantity of raw steel plate and product steel plate are given in Tables 1 and 2.

**Table 1:** Information of different kinds of products.

Number	Length/mm	Width/mm	Requirement (sheet)	Floating scale	Type
1	44,351.13	422.88	36	5%	Coil
2	39,229.01	282.88	29	–	Coil
3	54,787.74	268.36	42	–	Coil
4	45,284.39	277.7	32	1%	Coil
5	53,479.79	332.29	18	10%	Coil
6	897.32	603.06	38	–	Plate
7	896.09	714.72	23	10%	Plate
8	1096.33	435.84	31	10%	Plate
9	890.53	343.08	40	–	Plate
10	752.61	641.45	42	–	Plate
11	970.16	667.21	34	–	Plate
12	998.29	472.3	25	5%	Plate
13	1024.87	340.51	24	–	Plate
14	621.91	476.6	22	–	Plate
15	1243.03	471.25	28	10%	Plate

**Table 2:** Information of different kinds of raw materials.

Number	Length/mm	Width/mm	Stock (sheet)
1	148,623.91	1519.91	5
2	32,960.49	999.35	10
3	75,508.72	1232.32	8
4	14,091.52	920.62	2
5	75,040.31	1573.71	3
6	138,570.39	844.99	10
7	98,641.28	1184.54	12
8	114,074.27	879.02	9
9	104,637.72	969.02	3
10	58,023.82	1785.45	10

For all the available raw materials, our task is to use the minimum number of sheets to meet the requirements for all orders. At the same time, we attempt to minimize the number of tool changes and cuts on small machines, and maximize the total yield. The next section describes a mathematical model to solve the above problem.

## Problem analysis

Classical production problems often seek to maximize the overall raw material utilization and minimize the production costs. Labor costs (number of blade changes and number of workers operating small machines for processing) should also be considered. A model for multi-objective planning is now established. As the most intuitive optimization objective is the amount of raw materials used, the most important optimization objective is the number of sheets of raw steel plate required for the cutting plan. The secondary objective is the raw material yield, and the tertiary objective is the number of human operations.

## Model assumptions

1. Assume that there is no mechanical loss in the production and manufacturing process.
2. Assume that the float percentage only applies to the length of the product, rather than the width of the product.

## Model establishment

We first define several terms that are used in the remainder of the paper.

*Single column cutting scheme*: a cutting solution that uses one or more products lined up on the wide edge of a rectangular steel plate and can be cut using a one-cut process with the cutting shear. We start by arranging the same or different products on the wide edge of the raw material, packing them into a single column to optimize the combination, and aim to accomplish the task with very little raw material without using small machines. The single column cutting scheme contains information such as the amount of each raw material, the corresponding floating length range, and the number of products obtained from the column.

*Whole sheet cutting scheme*: a single sheet of a raw material with the single column cutting scheme results in a whole sheet cutting solution. The whole sheet cutting scheme contains information such as the serial number of the raw material to which the cut corresponds, information about the remaining material on the long edge, and the number of different products obtained from the scheme.

We wish to avoid the need for small machine cutting, instead using only the cutting shear to complete the processing task in the form of a one-cut. Therefore, for each raw material, after adjusting the floating ratio of different products to achieve a “floating equal length” (i.e., equal length of different rectangular products in the same single column cutting scheme), we need to obtain a single column cutting scheme for different product combinations. Single column cutting schemes can then be arranged on the long side of the raw material. To facilitate the description of the model, we present several symbols in Table 3.

**Table 3:** Symbol description.

Symbol	Description
$i$	Product number, *i* = 1…*NI*
*NI*	Maximum number of product types. From the data in Table 1, *NI* = 15
$j$	Material number, *j* = 1…*NJ*
*NJ*	Maximum number of types of raw materials. From the data in Table 2, *NJ* = 10
$WP_{i}$	Width of *i*-th product
$LP_{i}$	Length of *i*-th product
$WM_{j}$	Width of *j*-th material
$LM_{j}$	Length of *j*-th material
$NP_{i}$	Number of *i*-th product ordered
$k_{j}$	Single column cutting scheme number for *j*-th material
$Nk_{j}$	Total number of single column cutting schemes for *j*-th material
$NM_{j}$	Total inventory of *j*-th material
$h_{j}$	Serial number of *j*-th material used to process the product, *h*_*j*_ = 1…*NM*_*j*_
$PNP_{{ijk_{j} }}$	Product number of plan: quantity of *i*-th product obtained by cutting *k*_*j*_-th single column cutting scheme for *j*-th raw material
$NPU_{{jk_{j} }}$	Number of times *k*_*j*_-th single column cutting scheme is applied for *j*-th material
$LCP_{{jk_{j} }}$	Length of *k*_*j*_-th single column cutting scheme for *j*-th raw material
$NPM_{{jk_{j} h_{j} }}$	Number of times that *h*_*j*_-th sheet of *j*-th material is applied to *k*_*j*_-th single column cutting scheme
$SLM_{{jh_{j} }}$	Remaining length of long edge of *h*_*j*_-th sheet of *j*-th material being processed
$LS_{{jh_{j} }}$	Length of surplus material of *h*_*j*_-th sheet of *j*-th material being processed
$WS_{{jh_{j} }}$	Width of surplus material of *h*_*j*_-th sheet of *j*-th material being processed
$crP_{i}$	Length floating percentage of *i*-th product
$LP^{\prime}_{i}$	Actual length of *i*-th product
$Canjoin_{{i_{1} ,i_{2} }}$	0–1 variable used to indicate whether *i*_1_-th product and *i*_2_-th product can be equal in length by adjusting the floating ratio of product length; 1 for yes, 0 for no
$PIC_{{ijk_{j} }}$	0–1 variable used to determine whether *k*_*j*_-th single column cutting scheme has *i*-th product; 1 for yes, 0 for no
$NA_{{jh_{j} }}$	Number of blade changes for *h*_*j*_-th sheet of *j*-th material being processed
$NC$	Maximum number of disk blades that can be arranged at the same time. In our problem, *NC* = 5

### Establishment of the objective function

Our problem is a multi-objective planning problem with the goals of using the fewest sheets of raw material possible, achieving the highest yield possible, and minimizing the number of blade arrangements and small machine operations. In fact, we constrain the number of small machine operations to be zero, so the third goal is to minimize the number of blade arrangements.

*Objective function I*: Use the fewest sheets of raw materials possible. See formula (1).

1 $$ \min f_{1} = \sum\limits_{j = 1}^{NJ} {\sum\limits_{{h_{j} = 1}}^{{NM_{j} }} {\left\{ \begin{gathered} 1,\;if \,SLM_{{jh_{j} }} < LM_{j} \hfill \\ 0,\;if \,SLM_{{jh_{j} }} = LM_{j} \hfill \\ \end{gathered} \right.} } $$

When *SLM*_*jhj*_ = *LM*_*j*_, the *h*_*j*_-th sheet is not used. *SLM*_*jhj*_ < *LM*_*j*_ means that the *h*_*j*_-th sheet is used and needs to be counted in the total number of used sheets.

*Objective function II*: Maximize the overall yield. See formula (2).

2 $$ \max f_{2} = \frac{{\sum\limits_{i = 1}^{NI} {LP^{\prime}_{i} } \cdot WP_{i} + \sum\limits_{j = 1}^{NJ} {\sum\limits_{{h_{j} = 1}}^{{NM_{j} }} {LS_{{jh_{j} }} \cdot WS_{{jh_{j} }} } } }}{{\sum\limits_{j = 1}^{NJ} {\sum\limits_{{h_{j} = 1}}^{{NM_{j} }} {\left\{ \begin{gathered} LM_{j} \cdot WM_{j} ,\;if \,SLM_{{jh_{j} }} < LM_{j} \hfill \\ 0,\;if \,SLM_{{jh_{j} }} = LM_{j} \hfill \\ \end{gathered} \right.} } }} $$

where the denominator represents the total area of all used raw materials and the numerator represents the sum of the product area and the area that can be returned to storage.

*Objective function III*: Minimize the number of blade changes. See formula (3).

3 $$ \min f_{3} = \sum\limits_{j = 1}^{NJ} {\sum\limits_{{h_{j} = 1}}^{{NM_{j} }} {NA_{{jh_{j} }} } } $$

We use hierarchical optimization, which prioritizes the pursuit of the lowest possible number of sheets, followed by the pursuit of the highest possible total yield, followed by the pursuit of the lowest possible number of blade changes. This is used as the strategy for our solution.

### Global constraints

The entire processing task must be completed. Therefore, the following constraints can be imposed.

*Constraint 1*: The processed product must meet each order requirement. See formula (4).

4 $$ \sum\limits_{j = 1}^{NJ} {\sum\limits_{{k_{j} }}^{{Nk_{j} }} {NPU_{{jk_{j} }} } } \cdot PNP_{{ijk_{j} }} \ge NP_{i} \begin{array}{*{20}c} , & {i = 1...NI} \\ \end{array} $$

where *NPU* is the number of times the *k*_*j*_-th single column cutting scheme is applied for the *j*-th raw material.

*Constraint 2*: The number of sheets of each raw material must not exceed the inventory limit. See formula (5).

5 $$ \sum\limits_{{h_{j} = 1}}^{{NM_{j} }} {\left\{ \begin{gathered} 1,\;if\, SLM_{{jh_{j} }} < LM_{j} \hfill \\ 0,\;if \, SLM_{{jh_{j} }} = LM_{j} \hfill \\ \end{gathered} \right.} \le NM_{j} ,\,j = 1...NJ $$

### Constraint on single column cutting scheme

**Figure 7:**
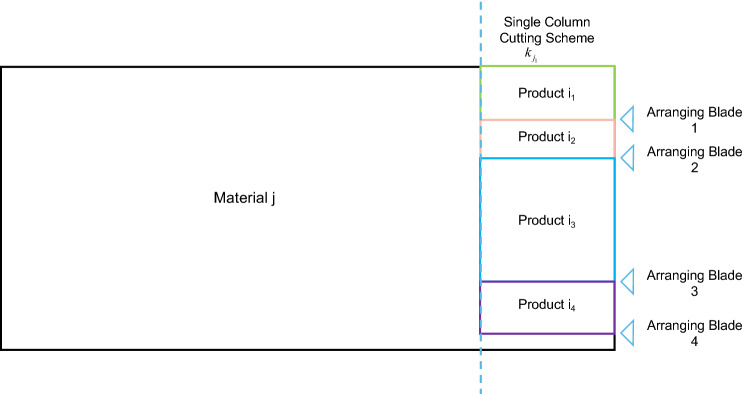
Schematic diagram of single column cutting scheme.

Single column widthwise packing (see Fig. 7) places the wide edge of different or the same products that have a floating equal length on the wide edge of a raw material for the maximum one-dimensional packing. By “maximum,” we mean that the remaining width of the raw material cannot be used for any other piece that can float with the same group of products. As shown in Fig. 7, each wide-directional packing method is recorded as a single column cutting scheme for that raw material. The total number of single row cutting solutions for one raw material and 15 products is *Nk*_*j*_, each cutting scheme number is *k*_*j*_, and the total number of products obtained for each cutting scheme is *PNP*_*ijk*_.

First, the products that be cut to an equal length by adjusting the float percentage are grouped together. Thus, we require a set of mathematical expressions to “group” all products that can be equal in length.

*Constraint 3*: Constraint on the range of product lengths that can be varied after adding the floating percentage. See formula (6).

6 $$ \begin{gathered} \min LP_{i}^{\prime } = (1 - crP_{i} )LP_{i} \hfill \\ \max LP_{i}^{\prime } = (1 + crP_{i} )LP_{i} \hfill \\ \min LP_{i}^{\prime } \le LP_{i}^{\prime } \le \max LP_{i}^{\prime } \hfill \\ i = 1...NI \hfill \\ \end{gathered} $$

where *LP*_*i*_*’* is the actual length of the *i*-th product, i.e., the length after adjusting the floating percentage.

*Constraint 4*: Add 0–1 variables to determine whether products *i*_1_ and *i*_2_ can be equal in length by adjusting the floating percentage; 1 for yes, 0 for no. See formula (7).

7 $$ \begin{gathered} Canjoin_{{i_{1} ,i_{2} }} = \left\{ \begin{gathered} 1, if\,\min LP^{\prime}_{{i_{1} }} \le \max LP^{\prime}_{{i_{2} }} and \min LP^{\prime}_{{i_{2} }} \le \max LP^{\prime}_{{i_{1} }} \hfill \\ 0, if\,\min LP^{\prime}_{{i_{1} }} > \max LP^{\prime}_{{i_{2} }} or \min LP^{\prime}_{{i_{2} }} > \max LP^{\prime}_{{i_{1} }} \hfill \\ \end{gathered} \right. \hfill \\ i_{1} = 1...NI,i_{2} = 1...NI \hfill \\ \end{gathered} $$

Once the grouping is complete, the next step is to sample the same group of products for each raw material in a wide-directional one-dimensional row, thus obtaining all the single column cutting schemes.

*Constraint 5*: Add a 0–1 variable to determine whether product *i* is present in scheme *k*_*j*_; 1 for yes, 0 for no. See formula (8).

8 $$ PIC_{{ijk_{j} }} = \left\{ \begin{gathered} 1,if\, PNP_{{ijk_{j} }} > 0 \hfill \\ 0,if \,PNP_{{ijk_{j} }} = 0 \hfill \\ \end{gathered} \right.,\begin{array}{*{20}c} {} & {j = 1...NJ,i = 1...NI} \\ \end{array} $$

*Constraint 6*: Multiple products that may exist in the same single column cutting scheme must have floating equal lengths. See formula (9).

9 $$ \begin{gathered} Canjoin_{{i_{1} ,i_{2} }} = 1,if\, PIC_{{i_{1} jk_{j} }} = PIC_{{i_{2} jk_{j} }} = 1 \hfill \\ j = 1...NJ,i_{1} = 1...NI,i_{2} = 1...NI \hfill \\ \end{gathered} $$

*Constraint 7*: A column cannot exceed the length of the wide side of the raw material (the remaining width cannot be negative), but the remaining width of the raw material cannot exceed the shortest wide side of the same group of products. This is the definition of maximum one-dimensional column packing. See formula (10).

10 $$ \begin{gathered} 0 \le WM_{j} - \sum\limits_{i = 1}^{NI} {PNP_{{ijk_{j} }} \cdot WP_{i} } < \min \{ WP_{i} |PIC_{{ijk_{j} }} = 1\} \hfill \\ j = 1...NJ \hfill \\ \end{gathered} $$

**Figure 8:**
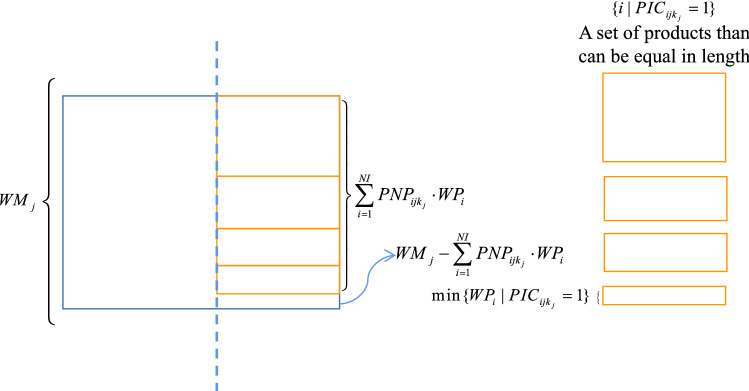
Diagram of maximum one-dimensional column packing.

The width of the remnants of a single column cutting scheme must be less than the smallest width of the same group of products (see Fig. 8).

*Constraint 8*: The number of products cut by a single column cutting scheme cannot exceed the maximum number of disc shears. See formula (11).

11 $$ \sum\limits_{i = 1}^{NI} {PNP_{{ijk_{j} }} \le NC} ,\begin{array}{*{20}c} {} & {} \\ \end{array} j = 1...NJ,k_{j} = 1...Nk_{j} $$

where *NC* represents the maximum number of disc shears that can be arranged at the same time. In our scenario, the maximum value is 5.

*Constraint 9*: The range of variation for the final length of the single column cutting scheme is the intersection of the floating length range of all products included in the scheme. That is, the final length must be greater than or equal to the highest possible minimum floating length of the same family of product species and less than or equal to the lowest possible maximum floating length of the same group of product species. See formula (12).

12 $$ \begin{gathered} \max \{ \min LP_{i}^{\prime } |PIC_{{ijk_{j} }} = 1\} \le LCP_{{jk_{j} }} \le \min \{ \max LP^{\prime}_{i} |PIC_{{ijk_{j} }} = 1\} \hfill \\ j = 1...NJ,i = 1...NI \hfill \\ \end{gathered} $$

where *LCP*_*jk*_ is the actual length of the *k*_*j*_-th single column cutting scheme for the *j*-th material.

In this way all the single column cutting models can be determined from the above constraints. Next, these single column cutting schemes are applied to each raw material for long-edge one-dimensional row packing.

### Constraint on the whole sheet of raw material

**Figure 9:**
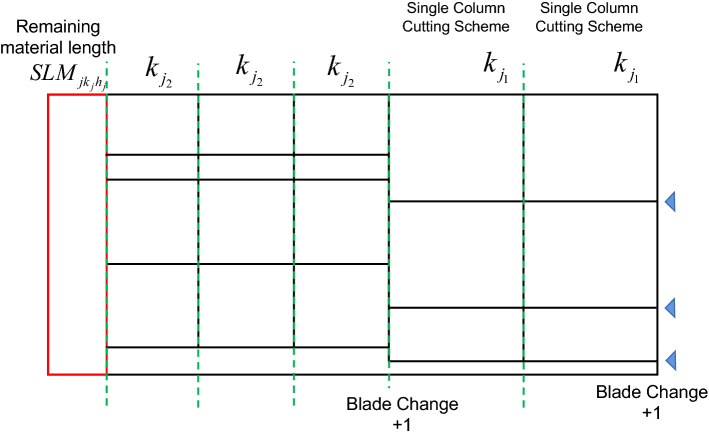
Schematic diagram of whole sheet cutting scheme.

The material is lined up on the long edge using a single column cutting scheme (as shown in Fig. 9, the whole sheet cutting scheme is the combination of multiple single column cutting schemes). In practice, the cutting of each column is carried out with a one-cut process by the cutting shear, without additional small machine processing. This approach necessarily gives **zero small machine operations**, but it is difficult to avoid the generation of scrap, i.e., the bottom edge of each single column cutting scheme. In addition, there is no need to change the row of blades between the same single column cutting schemes. The blades are only rearranged when the single column cutting scheme is changed. Thus, the number of blade changes is equal to the number of different single column cutting schemes.

*Constraint 10*: The residual length of each sheet of raw material after the long-directional row of samples is calculated as follows: See formula (13).

13 $$ \begin{gathered} SLM_{{jh_{j} }} = LM_{j} - \sum\limits_{{k_{j} = 1}}^{{Nk_{j} }} {NPM_{{jk_{j} h_{j} }} \cdot LCP_{{jk_{j} }} } \hfill \\ j = 1...NJ,h_{j} = 1...NM_{j} \hfill \\ \end{gathered} $$

where *LCP*_*jk*_ is the length of the *k*_*j*_-th single column cutting scheme for the *j*-th raw material; *NPM*_*jkh*_ is the number of times the *k*_*j*_-th single column cutting scheme is applied to the *h*_*j*_-th sheet of the *j*-th raw material; and *LM*_*j*_ is the original length of the *j*-th raw material.

*Constraint 11*: The length of a single sheet of material cannot exceed the long-direction packing (i.e., the remaining length cannot be negative). See formula (14).

14 $$ SLM_{{jh_{j} }} \ge 0\begin{array}{*{20}c} , & {} \\ \end{array} j = 1...NJ,h_{j} = 1...NM_{j} $$

*Constraint 12*: The number of changes to the row of blades for cutting a single sheet of material is calculated by formula (15).

15 $$ NA_{{jh_{j} }} = \sum\limits_{{k_{j} = 1}}^{{Nk_{j} }} {\left\{ \begin{gathered} 1,if \,NPM_{{jk_{j} h_{j} }} > 0 \hfill \\ 0,if\, NPM_{{jk_{j} h_{j} }} = 0 \hfill \\ \end{gathered} \right.} ,j = 1...NJ,h_{j} = 1...NM_{j} $$

where *NPM*_*jkh*_ is the number of times the *h*_*j*_-th sheet of the *j*-th raw material is used for the *k*_*j*_-th single column cutting scheme.

*Constraint 13*: The effective residual material after the long-direction packing is given by formula (16).

16 $$ \begin{gathered} LS_{{jh_{j} }} = \left\{ \begin{gathered} SLM_{{jh_{j} }} ,if \,SLM_{{jh_{j} }} \ge 2000\, and \,WM_{j} \ge 1000 \hfill \\ 0,if \,SLM_{{jh_{j} }} < 2000 \,or\, WM_{j} < 1000 \hfill \\ \end{gathered} \right. \hfill \\ WS_{{jh_{j} }} = \left\{ \begin{gathered} WM_{j} ,if\, SLM_{{jh_{j} }} \ge 2000 \,and\, WM_{j} \ge 1000 \hfill \\ 0,if \,SLM_{{jh_{j} }} < 2000\, or \,WM_{j} < 1000 \hfill \\ \end{gathered} \right. \hfill \\ j = 1...NJ,h_{j} = 1...NM_{j} \hfill \\ \end{gathered} $$

After completing a long-direction packing, the final remaining long-edge remnant may be recyclable. Thus, the length and width are determined. If the remnant meets the return requirements, the length and width are recorded. If the remnant does not meet the return requirement, the length and width of the remaining material are set to zero, indicating that there is no valid returnable material.

The proposed mathematical model can be summarized as follows:

$$ \min f_{1} = \sum\limits_{j = 1}^{NJ} {\sum\limits_{{h_{j} = 1}}^{{NM_{j} }} {\left\{ {\frac{{1,\;if\, SLM_{{jh_{j} }} < LM_{j} }}{{0,\;if \,SLM_{{jh_{j} }} = LM_{j} }}} \right.} } $$

$$ \max f_{2} = \frac{{\sum\limits_{i = 1}^{NI} {LP_{i}^{^{\prime}} \cdot } WP_{i} + \sum\limits_{j = 1}^{NJ} {\sum\limits_{{h_{j} = 1}}^{{NM_{j} }} {LS_{{jh_{j} }} \cdot WS_{{jh_{j} }} } } }}{{\sum\limits_{j = 1}^{NJ} {\sum\limits_{{h_{j} = 1}}^{{NM_{j} }} {\left\{ \begin{gathered} LM_{j} \cdot WM_{j} ,\;if \,SLM_{{jh_{j} }} < LM_{j} \hfill \\ 0,\;if \,SLM_{{jh_{j} }} = LM_{j} \hfill \\ \end{gathered} \right.} } }} $$

$$ \min f_{3} = \sum\limits_{j = 1}^{NJ} {\sum\limits_{{h_{j} = 1}}^{{NM_{j} }} {NA_{{jh_{j} }} } } $$

$$ s.t.\left\{ \begin{gathered} \sum\limits_{j = 1}^{NJ} {\sum\limits_{{k_{j} }}^{{Nk_{j} }} {NPU_{{jk_{j} }} } } \cdot PNP_{{ijk_{j} }} \ge NP_{i} \begin{array}{*{20}c} , & {i = 1...NI} \\ \end{array} \hfill \\ \sum\limits_{{h_{j} = 1}}^{{NM_{j} }} {\left\{ \begin{gathered} 1,\;if\, SLM_{{jh_{j} }} < LM_{j} \hfill \\ 0,\;if \,SLM_{{jh_{j} }} = LM_{j} \hfill \\ \end{gathered} \right.} \le NM_{j} ,\;j = 1...NJ \hfill \\ \min LP_{i}^{\prime } = (1 - crP_{i} )LP_{i} \hfill \\ \max LP_{i}^{\prime } = (1 + crP_{i} )LP_{i} \hfill \\ \min LP_{i}^{\prime } \le LP_{i}^{\prime } \le \max LP_{i}^{\prime } \hfill \\ Canjoin_{{i_{1} ,i_{2} }} = \left\{ \begin{gathered} 1, if\,\min LP^{\prime}_{{i_{1} }} \le \max LP^{\prime}_{{i_{2} }} \,and\, \min LP^{\prime}_{{i_{2} }} \le \max LP^{\prime}_{{i_{1} }} \hfill \\ 0, if\,\min LP^{\prime}_{{i_{1} }} > \max LP^{\prime}_{{i_{2} }}\, or \,\min LP^{\prime}_{{i_{2} }} > \max LP^{\prime}_{{i_{1} }} \hfill \\ \end{gathered} \right. \hfill \\ PIC_{{ijk_{j} }} = \left\{ \begin{gathered} 1,if \,PNP_{{ijk_{j} }} > 0 \hfill \\ 0,if\, PNP_{{ijk_{j} }} = 0 \hfill \\ \end{gathered} \right.,\begin{array}{*{20}c} {} & {j = 1...NJ,i = 1...NI} \\ \end{array} \hfill \\ Canjoin_{{i_{1} ,i_{2} }} = 1,if\, PIC_{{i_{1} jk_{j} }} = PIC_{{i_{2} jk_{j} }} = 1 \hfill \\ 0 \le WM_{j} - \sum\limits_{i = 1}^{NI} {PNP_{{ijk_{j} }} \cdot WP_{i} } < \min \{ WP_{i} |PIC_{{ijk_{j} }} = 1\} \hfill \\ \sum\limits_{i = 1}^{NI} {PNP_{{ijk_{j} }} \le NC} ,\;j = 1...NJ,k_{j} = 1...Nk_{j} \hfill \\ \max \{ \min LP_{i}^{\prime } |PIC_{{ijk_{j} }} = 1\} \le LCP_{{jk_{j} }} \le \min \{ \max LP^{\prime}_{i} |PIC_{{ijk_{j} }} = 1\} \hfill \\ SLM_{{jh_{j} }} = LM_{j} - \sum\limits_{{k_{j} = 1}}^{{Nk_{j} }} {NPM_{{jk_{j} h_{j} }} \cdot LCP_{{jk_{j} }} } ,\;j = 1...NJ,h_{j} = 1...NM_{j} \hfill \\ SLM_{{jh_{j} }} \ge 0,\;j = 1...NJ,h_{j} = 1...NM_{j} \hfill \\ NA_{{jh_{j} }} = \sum\limits_{{k_{j} = 1}}^{{Nk_{j} }} {\left\{ \begin{gathered} 1,if \,NPM_{{jk_{j} h_{j} }} > 0 \hfill \\ 0,if \,NPM_{{jk_{j} h_{j} }} = 0 \hfill \\ \end{gathered} \right.} ,j = 1...NJ,h_{j} = 1...NM_{j} \hfill \\ LS_{{jh_{j} }} = \left\{ \begin{gathered} SLM_{{jh_{j} }} ,if \,SLM_{{jh_{j} }} \ge 2000 \,and\, WM_{j} \ge 1000 \hfill \\ 0,if \,SLM_{{jh_{j} }} < 2000 \,or \,WM_{j} < 1000 \hfill \\ \end{gathered} \right. \hfill \\ WS_{{jh_{j} }} = \left\{ \begin{gathered} WM_{j} ,if\, SLM_{{jh_{j} }} \ge 2000\, and \,WM_{j} \ge 1000 \hfill \\ 0,if\, SLM_{{jh_{j} }} < 2000\, or \,WM_{j} < 1000 \hfill \\ \end{gathered} \right. \hfill \\ \end{gathered} \right. $$

## Algorithm details

For the multi-objective planning problem considered in this study, we have developed an algorithm for determining all single column cutting solutions and a real number coding-based genetic algorithm for the production problem^14–16^. The four-step process is described below.

### Step 1: product grouping

The grouping is based on our definition of the floating equal length, i.e., the products can be made equal in length by adjusting the floating percentage of the products. All products that can be equal in length are grouped so that products from the same group can be scheduled along the wide direction of the single column cutting scheme.

**Figure 10:**
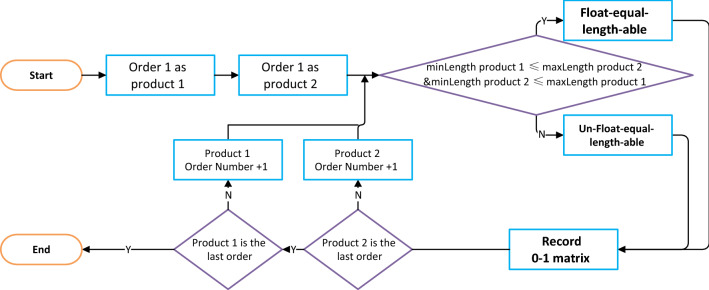
Flowchart of product grouping algorithm.

Using a simple arithmetic process (see Fig. 10), we construct a 0–1 matrix to determine whether any two products have a floating equal length, i.e., corresponding to *Canjoin*_*i1,i2*_ in the model. After the grouping is finished, a search is performed to find all single column cutting schemes.

### Step 2: single column cutting scheme search

**Figure 11:**
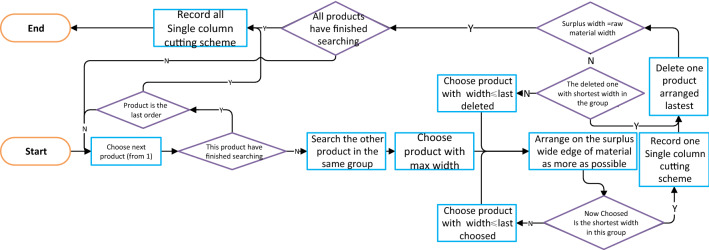
Flowchart of single column cutting scheme searching algorithm.

The single column cutting scheme search algorithm proceeds according to the flowchart in Fig. 11. According to the order of selecting the longest first and then the shorter, until after selecting the shortest products on the wide edge, delete the products arranged at the end in turn, until after deleting a product that is not the shortest width, and then select the products with the next widest width in order to line up the sample as much as possible, so that a single column cutting scheme can be found without missing.

### Step 3: whole sheet cutting search

**Figure 12:**
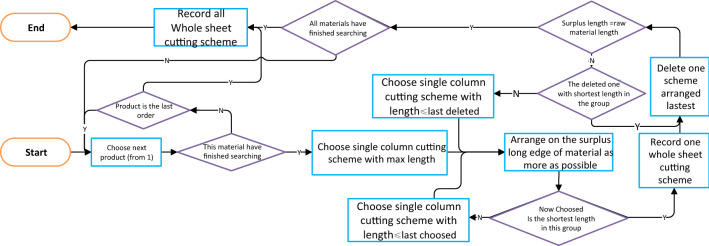
Flowchart of whole sheet cutting scheme search algorithm.

Step 3 proceeds as shown in Fig. 12. This step involves additional work not described in the mathematical model, and the solution needs to be converted into a form whereby the constraints can be discriminated to facilitate the subsequent use of intelligent algorithms. If the solution is described only in terms of a single column cutting scheme, it is difficult to judge whether the number of sheets of raw material is suitable for a certain number of single column cutting schemes. However, if converted to a cutting scheme for a whole sheet of raw material, it is much less difficult to determine whether the solution satisfies the constraint.

The whole sheet cutting schemes obtained from this step contain information on the type of raw material corresponding to each scheme, the quantity of the various products obtained, and the length and width of the residual material.

### Step 4: main program and auxiliary search solution operators

We use a heuristic algorithm to solve this mathematical model. General iterative methods cannot be used because this is a high-dimensional mixed-integer nonlinear programming model, and so the number of iterations would approach infinity. Instead, a global optimization search must be performed strategically.

Genetic algorithms are computational models of biological evolution, simulating the natural selection and genetic mechanisms found in the natural world to identify optimal solutions^17,18^. One problem encountered by intelligent algorithms in solving optimization-like problems is the difficulty in coping with mathematical models with many constraints^19^. In the case of downscaling problems, there are constraints on raw materials and on products, and the occurrence of invalid or infeasible solutions significantly reduces the efficiency of the intelligent algorithm.

**Figure 13:**
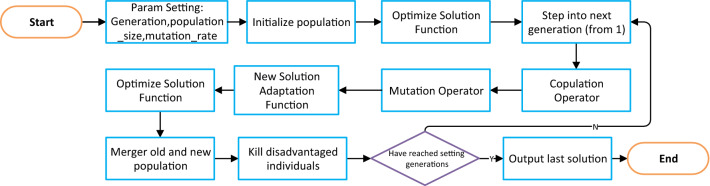
Flowchart of innovative genetic algorithm with real number encoding.

In this study, we design special genetic algorithms for production class problems. A real number encoding approach is used as an innovative means of expressing the solution. Figure 13 shows a flowchart of the main loop of the genetic algorithm.

**Figure 14:**
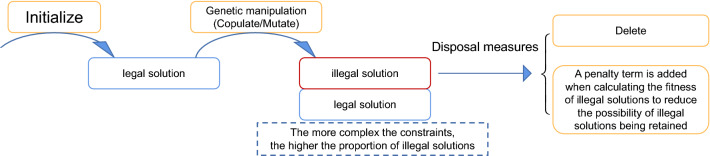
Traditional genetic algorithm search process for solutions.

Traditional genetic algorithms implement classical genetic operations such as crossover and mutation. This introduces a large degree of randomness to the variation of individual solutions, which can easily result in invalid solutions that do not satisfy the constraints. In general, solutions that do not meet the constraints are removed or have a large penalty term added to their fitness values, thus reducing their ability to compete with legitimate solutions (see Fig. 14). In the case of complex constraints, however, the probability of valid solutions being generated on each iteration is very low, constituting a waste of computational power.

**Figure 15:**
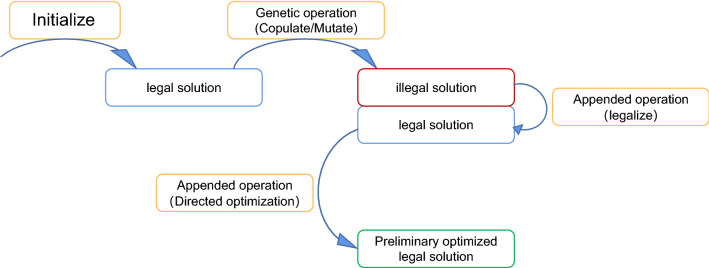
Improved genetic algorithm search process for solutions.

To overcome this problem, we improve the framework of the traditional genetic algorithm by adding a step to validate and optimize new solutions in a directed manner (see Fig. 15). The validation improves the rate of valid new solutions to 100%, while the optimization ensures the superiority of new solutions. These two operations lengthen the runtime of each genetic cycle, but improve the speed with which superior solutions are identified.

We conducted experiments to verify the efficiency of these validation and optimization steps. Without the validation step, the algorithm generated 400 new solutions in 3.85 s, of which only three were valid. Adding the validation step increased the time taken to generate 400 solutions to 25.96 s, but all 400 solutions were valid. Thus, it takes 1.28 s to generate a legal solution before adding the validation step, and only 0.0649 s to generate a legal solution after adding the validation step, a factor of 20 improvement.

**Figure 16:**
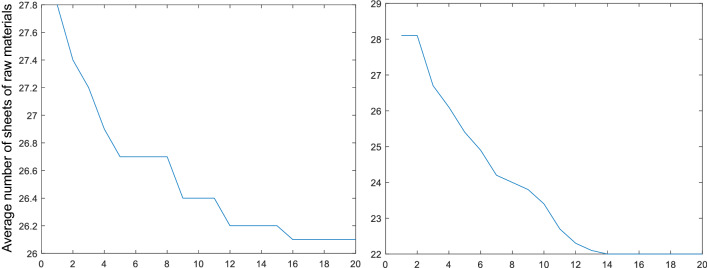
Optimization curves of fitness without (left) and with (right) targeted optimization.

Next, we examined the quality of the solutions before and after adding the directed optimization algorithm. The optimization curves over 20 genetic iterations are compared in Fig. 16.

The convergence speed increases significantly after adding the directional optimization step, and a higher quality of solution is achieved. These results prove that this modification is effective.

**Figure 17:**
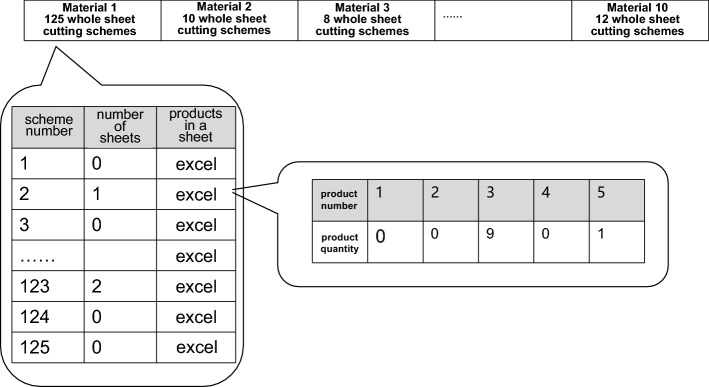
Expressions of the solution.

Before describing the initial solution functions, we present the data structure of the individual solutions to facilitate the subsequent explanation. Figure 17 shows the detailed phenotype of the solutions.

The variable to be determined is the number of sheets of each raw material required for the whole cutting solution. Each solution corresponds to the production of different products, and the sum of the sheets over all solutions is the number of sheets of raw material consumed. With this description of the solution, the crossover and mutation operations are simply a matter of applying the tensor to the solution. The functions and implementation methods of each auxiliary function in the genetic algorithm are described below.

**Figure 18:**
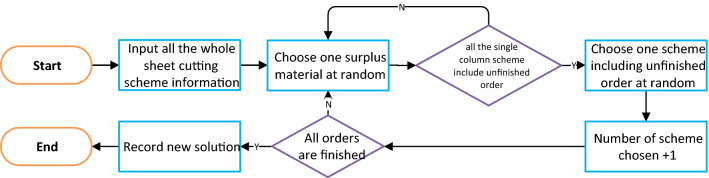
Flowchart of initialization function of genetic algorithm.

The initial solution function (see Fig. 18) generates a random valid solution. This randomly generated valid solution satisfies all constraints. It is difficult to achieve good optimization with purely randomly generated solutions, but an arbitrary initial population of valid solutions is needed for comparison with subsequently generated solutions.

The individuals given by the initial solution function are not optimized. In particular, there are some whole-cutting solutions that are completely redundant, i.e., even when they are subtracted, the machining task can still be completed without breaking the constraints. Therefore, the initially generated solutions require some preliminary optimization.

**Figure 19:**
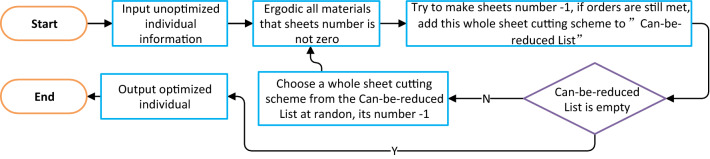
Flowchart of optimal solution function of genetic algorithm.

The optimal solution function (see Fig. 19) achieves individual fitness optimization in a relatively straightforward way. Recall that the minimum number of raw material sheets used is the measure of the fitness of the solution, so the status of this function is quite important.

The optimal solution function modifies the initial solution such that reducing any of the products will cause the solution to violate a constraint. However, it is difficult to find the global optimal solution by adjusting the solution in this way. The core parts of the genetic algorithm come from offspring generation and mutation.

**Figure 20:**
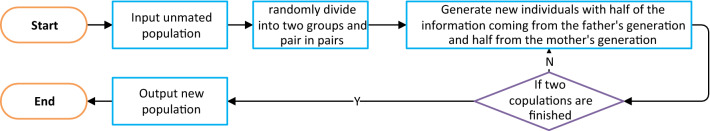
Flowchart of offspring generation in genetic algorithm.

Offspring generation involves crossing the chromosomes of two parent solutions. Two individuals from the original population are selected as the parents and combined to produce two offspring. The fitness of these two offspring may be better or worse than that of the parents, but once the parents have identified the dominant gene, the offspring inherit this gene, ensuring a solution with good fitness. The offspring generation function is instrumental in allowing the genetic algorithm to search for solutions efficiently. A flowchart of this procedure is shown in Fig. 20.

**Figure 21:**
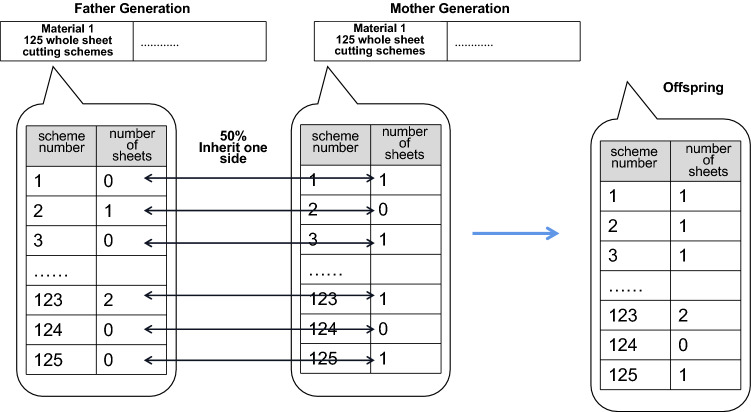
Schematic diagram of offspring generation.

The offspring generation function is illustrated in Fig. 21. Tensor selection is applied to one side of the corresponding solutions of the parents, and the resulting offspring contain the genes of both parents. However, the newly generated offspring is not necessarily a valid solution. In the offspring solution in Fig. 21, the number of applied sheets has reached 6, while the total number available is only 5. Thus, the solution violates a constraint. The probability of a valid solution being generated is low. However, directly deleting the solution and generating more offspring may result in a long series of invalid solutions. Instead, we design multiple functions that adjust the individual solutions.

**Figure 22:**
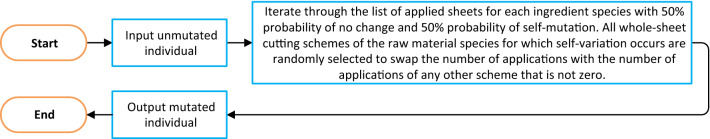
Flowchart of mutation function of genetic algorithm.

Before introducing the adjustment solution function, we introduce the mutation function. This function prevents the algorithm from becoming trapped around a locally optimal solution. As the solution process gradually converges, all the genes gradually converge. In generating offspring, highly similar genes in both parents will produce almost identical genes in the new offspring. If the population remains near a locally optimal solution, it will not be able to break away and explore the solution space. Thus, it is necessary to generate mutations that are not present in the original population. A flowchart of the mutation operation is shown in Fig. 22.

The new offspring, with or without individual mutations, do not necessarily satisfy all constraints. To improve the efficiency of these new individuals and the performance of the algorithm, a fine-tuning step is required.

**Figure 23:**
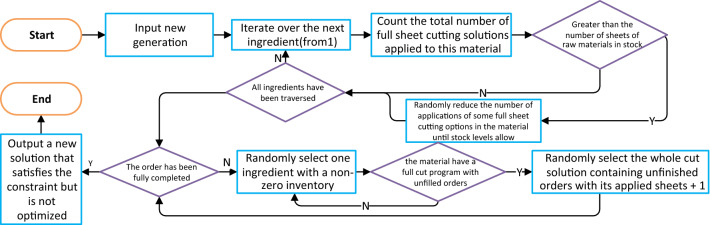
Flowchart of new solution adaptation function of genetic algorithm.

The new solution adaptation function adjusts invalid solutions to give valid solutions. This function is divided into two parts: the first part detects whether the upper limit of raw material usage has been exceeded and performs a deletion process, while the second part detects whether the order demand is satisfied and performs an addition process. A flowchart of this function is shown in Fig. 23.

As the new solution may only satisfy the constraints after the adaptation step and is unlikely to be optimal, the optimized solution function is applied once more to improve the solution.

The offspring population is merged with the parent population and the fitness values of all individuals are calculated. To prevent the population from becoming too large, half of the individuals are eliminated. The culling is not random, but instead removes the half of the individuals with the worst fitness values. In this way, the average fitness of the new population is necessarily equal to or better than the fitness of the original population.

Higher fitness values in the population imply a higher probability of the offspring integrating the superior genes of the parents. Once the fitness converges to a certain value, the limit is broken by applying a mutation operator with a small probability. Once a better individual has been produced, it is retained and produces offspring with other individuals, enhancing the overall fitness of the population.

## Solving the model

Multi-objective planning is requires a comprehensive consideration of all objectives when performing integrated optimization. Multi-objective optimization models are generally solved using one of the following methods^20^.

*Hierarchical Sequence Method*: applicable to cases where there is an obvious order of priority between the objective functions. Higher-priority objectives are pursued at the expense of lower-priority objectives.

*Linear Weighting Method*: used where the importance of each objective function can be effectively quantified. First, the objective function values are normalized, weighted, and summed according to the importance of each objective function. If the importance cannot be accurately quantified, evaluation methods such as hierarchical analysis can be used to assess the importance of each objective function and calculate the importance weights.

*Ɛ-Constraint Method*: applicable where there is a significant primary and secondary relationship among the objectives and the secondary objectives are clearly delimited. This method first selects one of the objectives for optimization and transforms the remaining objectives into constraints by adding bounds. For a minimization objective, an upper bound is added as a constraint; for a maximization objective, a lower bound is added as a constraint.

*Pareto Method*: applicable to each objective function, but does not provide a good measure of the importance of each objective. The objective functions can have some mutual influence on each other, but may also be completely independent. This method eventually gives a Pareto-optimal solution set, in which the optimal solution of all the objective functions is recorded and dominated solutions are not recorded. A dominated solution has no objective function whose value is better than another solution in the Pareto-optimal solution set.

*Return Value-based Method*: useful when there is a deeper common goal among the objectives. This method requires an in-depth portrayal of the essential requirements of each objective function. For example, when pursuing multiple objectives such as the minimum total distance of transportation and minimum number of trips in a cargo transportation plan, multiple objectives can be grouped into a single objective of “transportation cost” by querying the relevant data.

Our mathematical model includes three objectives: minimize the number of rectangular raw materials used, maximize the raw material utilization, and minimize the number of blade changes. We solve this multi-objective planning model using the Pareto method. In the hierarchical sequence method, linear weighting method, and Ɛ-constraint method, the importance of different objectives is difficult to compare. In the return value-based method, it is impossible to determine the cost relationship between raw materials and labor consumption. The main reason for choosing the Pareto method is that the objective functions do not exist in isolation from each other, but they are not significantly correlated either. In minimizing the number of rectangular raw materials used, while maximizing the rate of raw materials, the cutting process to produce as little waste as possible naturally increases the utilization rate and reduces the number of sheets of raw material. However, by producing as little residual material as possible, the utilization rate increases, but the amount of material used does not decrease. In practice, it is not reasonable to make incomplete cuts on every sheet. The objective of minimizing the number of blade changes can be achieved by using the same single column cutting scheme as many times as possible on the same piece of material. However, using as few cutting solutions as possible may generate more scrap, which will negatively affect the other two objective functions. Therefore, the three objective functions cannot be optimized simultaneously. The Pareto method continuously updates the optimal solutions, retains the Pareto-optimal solution set, and outputs different optimal solutions. Finally, the optimal solution is determined according to the actual machining task.

**Figure 24:**
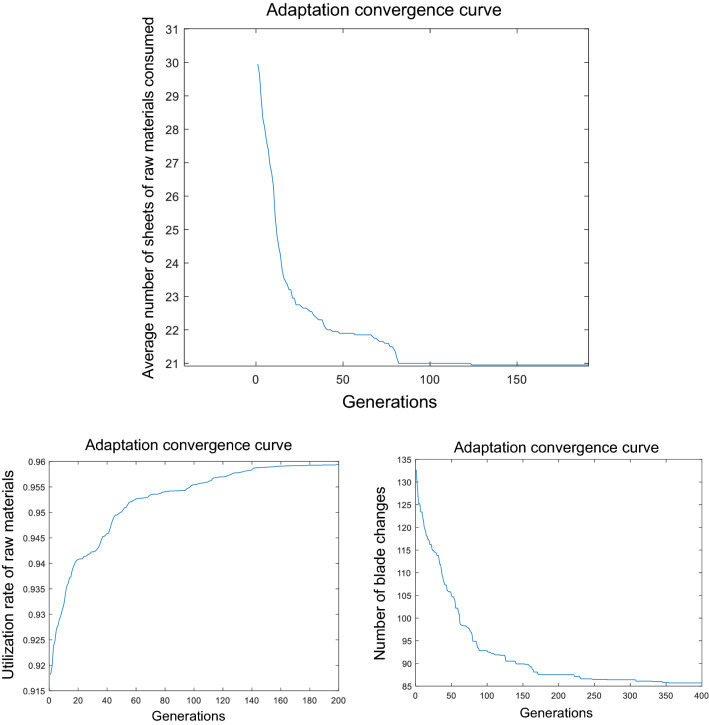
Genetic fitness curves.

The proposed optimization model was executed using the Matlab genetic algorithm main solver. The evolution of the population mean of the three objective functions is shown in Fig. 24. In the Pareto method, the convergence is not uniform, so the fitness curves are shown separately.

The results converge to 21 sheets of raw material after around 100 generations. A mutant individual generated after around 20,000 generations breaks through to 20 sheets of raw materials, giving the final optimal solution.

Table 4 presents the set of Pareto optimal solutions obtained after the final operation. The advantages and disadvantages of each solution are very eclectic and cannot be compared quantitatively. In practice, an optimal cutting solution can be identified according to the actual cost situation.

**Table 4:** Pareto-optimal solution set.

Scheme number	Sheets of raw material	Total yield	Total number of blade changes
1	20	0.9275816	115
2	20	0.9273717	114
3	20	0.9273255	112
4	20	0.9267204	111
5	23	0.9296370	121
6	20	0.9244498	108
7	22	0.9264080	104
8	22	0.9307681	125
9	24	0.9318606	113
10	30	0.9337798	132
11	20	0.9246980	110

We consider processing solution 3 (20 sheets of raw material consumed, 92.73% yield, 112 blade changes) as the optimal compromise solution. Table 5 presents a partial breakdown of this result, where the first column shows the amount of raw materials used and columns 2–16 show the number of products 1–15 obtained, respectively. The details of the cutting scheme are not easy to display here. Full solution results and the Matlab model solver are given in Annex I.

**Table 5:** Sample result.

Material number	P1	P2	P3	P4	P5	P6	P7	P8	P9	P10	P11	P12	P13	P14	P15	Utilization	Blade changes
1	5	0	5	2	0	0	1	0	0	2	0	1	0	0	0	92.08%	5
1	3	5	4	0	1	0	2	5	6	0	1	4	1	0	2	91.76%	10
1	3	0	4	5	1	2	1	1	0	0	3	0	0	0	2	91.25%	7
1	3	0	3	5	2	0	0	0	0	0	0	0	0	0	0	90.81%	3
1	4	0	1	4	3	0	0	9	0	0	2	0	0	0	0	94.24%	5
5	0	0	4	0	1	3	7	1	12	2	6	2	0	3	2	95.86%	11
5	0	0	3	0	2	13	7	0	0	6	12	2	0	3	3	92.61%	9
5	3	0	0	1	0	15	4	0	0	24	3	0	0	15	0	93.29%	7
6	0	0	6	0	0	2	1	5	10	1	0	4	10	1	2	86.72%	11
7	0	4	4	0	0	0	0	0	0	0	0	0	0	0	0	98.22%	2
7	0	4	3	0	1	0	0	0	0	0	0	0	0	0	0	95.64%	2
7	0	4	1	0	2	0	0	0	0	0	0	0	0	0	0	97.48%	2
7	0	4	0	0	3	0	0	0	0	0	0	0	0	0	0	97.10%	2
7	4	0	0	2	0	1	0	1	3	1	3	3	0	0	5	90.67%	7
7	3	0	0	3	0	0	0	0	0	0	0	0	0	0	0	89.54%	2
7	2	0	0	5	0	0	0	0	0	0	0	0	0	0	0	95.02%	2
7	2	4	0	1	0	0	0	4	3	4	0	0	0	0	10	87.82%	6
7	0	4	0	4	0	0	0	3	3	0	3	4	5	0	1	94.40%	8
8	2	0	2	0	1	2	0	2	4	2	0	5	6	0	0	93.11%	9
9	2	0	2	0	1	0	0	1	0	0	1	0	2	0	1	90.72%	5

**Figure 25:**
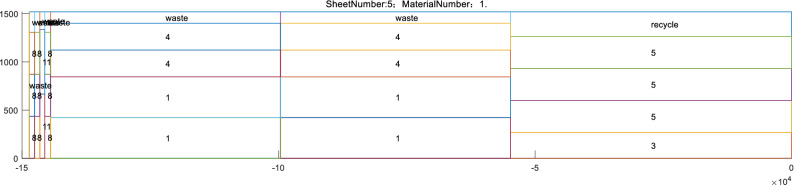
Rectangular packing result chart.

**Figure 26:**
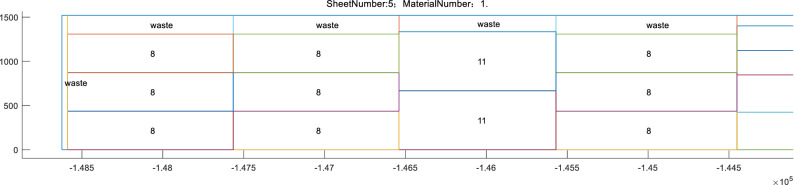
Cutting detail of the plate products.

Figures 25 and 26 show one of the rectangular packing results output by the Matlab program. A Matlab program and a visual plotting function are given in the annex, allowing more detailed nesting results to be viewed.

Note that the long side of the whole raw material cutting effect shown in Fig. 25 is compressed. This is because the raw material considered herein is a steel coil, which is very long and difficult to display here. Products 1–5 are of the “coil” type, which are also better displayed in a compressed chart. The numbers in the diagram correspond to the product number, “waste” indicates cutting waste, and “recycle” is surplus material that can be returned to storage. The denser rectangles on the left are the compressed “plate” type products, i.e., products 6–15, which appear to be compressed because they are smaller than the other pieces. Enlarging the area on the left yields Fig. 26, which shows the cutting detail of the plate products.

## Discussion and analysis

The research described in this paper has focused on a special cutting method and the associated mathematical model, as well as an innovative genetic algorithm for this cutting method. The advantages and disadvantages of the model and algorithm are discussed below.

The proposed mathematical model is mainly designed mainly for circular sawing machines, and the cutting solutions designed by this model are applicable to many different cutting processes, such as laser cutting machines. The model can also be applied to the design of raw material packing solutions of various sizes, so the generality and universality of our approach are very strong. The mathematical model is also designed to transform the two-dimensional packing problem into two one-dimensional packing problems, which essentially simplifies the mathematics, simplifies the calculation process, and improves the solution speed in the case of large amounts of data and large numbers of rectangular pieces.

Although the model has good generalizability, the optimization effect could be improved. For cutting tasks with normal dimensions and a small number of rectangular parts or for scheduling tasks, a high degree of optimization is rarely achieved. The nesting of a small number of rectangular parts has been extensively studied. Our approach is more targeted to the problem of large numbers of rectangular pieces, as encountered in actual cutting operation tasks.

The advantages of our algorithm are that the efficiency of new solutions is greatly improved, and it is possible to find new solutions with better merit in a directional way. This significantly reduces the randomness of the heuristic search for valid solutions, and enables very good solutions to be rapidly identified.

The main disadvantage of our algorithm is that the global nature of the search solution is reduced. The improvements to the algorithm mainly move the region of the heuristic search closer to the region of the clearly characterized superior solution. This strengthens the performance of solutions given by the genetic algorithm in a short period of time, but ignores some regions of the solution space that are not clearly characterized. Our approach may not perform better than a traditional genetic algorithm in relatively small-scale problems. Of course, actual production problems often involve large-scale, complex data, for which an improved genetic algorithm is more practical.

## Conclusions

This paper has presented a modeling analysis of the cutting problem in steel processing. The proposed model is highly practical due to the use of a pure cutting shear, which optimizes the utilization rate and the number of sheets of raw material with greatly reduced manpower requirements. The final result is a cutting solution with approximately 92.73% utilization and minimal process complexity. This model may be applicable in the planning of long steel processing. Appropriate programs and intelligent systems that provide real-time guidance in steel processing plants can greatly improve the efficiency of steel production and significantly reduce production costs.

## Supplementary Information

Supplementary Information 1.

Supplementary Information 2.

## Data Availability

All data are available and originate from real industrial production problems. The data provided in this paper are available directly and free of charge in addition to Tables 1 and 2, as are the detailed cutting production scenarios for the final program planning given in Annex I. In addition, the source code of the algorithm is provided in Annex II for download. None of the authors have a conflict of interest. The first author independently conducts research design and result analysis, and the corresponding author has a key contribution to the provision of data.
